# Rapid screening of secondary aromatic metabolites in *Populus trichocarpa* leaves

**DOI:** 10.1186/s13068-023-02287-2

**Published:** 2023-03-10

**Authors:** Anne E. Harman-Ware, Madhavi Z. Martin, Nancy L. Engle, Crissa Doeppke, Timothy J. Tschaplinski

**Affiliations:** 1 Renewable Resources and Enabling Sciences Center, National Renewable Energy Laboratory, Golden, CO 80401 USA; 2 Biosciences Division, Center for Bioenergy Innovation, Oak Ridge National Laboratory, Oak Ridge, TN 37831 USA

**Keywords:** Pyrolysis-molecular beam mass spectrometry, *Populus trichocarpa*, High-throughput analysis, Metabolomics

## Abstract

**Background:**

High-throughput metabolomics analytical methodology is needed for population-scale studies of bioenergy-relevant feedstocks such as poplar (*Populus* sp.). Here, the authors report the relative abundance of extractable aromatic metabolites in *Populus trichocarpa* leaves rapidly estimated using pyrolysis-molecular beam mass spectrometry (py-MBMS). Poplar leaves were analyzed in conjunction with and validated by GC/MS analysis of extracts to determine key spectral features used to build PLS models to predict the relative composition of extractable aromatic metabolites in whole poplar leaves.

**Results:**

The Pearson correlation coefficient for the relative abundance of extractable aromatic metabolites based on ranking between GC/MS analysis and py-MBMS analysis of the Boardman leaf set was 0.86 with *R*^2^ = 0.76 using a simplified prediction approach from select ions in MBMS spectra. Metabolites most influential to py-MBMS spectral features in the Clatskanie set included the following compounds: catechol, salicortin, salicyloyl-coumaroyl-glucoside conjugates, α-salicyloylsalicin, tremulacin, as well as other salicylates, trichocarpin, salicylic acid, and various tremuloidin conjugates. Ions in py-MBMS spectra with the highest correlation to the abundance of extractable aromatic metabolites as determined by GC/MS analysis of extracts, included *m/z* 68, 71, 77, 91, 94, 105, 107, 108, and 122, and were used to develop the simplified prediction approach without PLS models or a priori measurements.

**Conclusions:**

The simplified py-MBMS method is capable of rapidly screening leaf tissue for relative abundance of extractable aromatic secondary metabolites to enable prioritization of samples in large populations requiring comprehensive metabolomics that will ultimately inform plant systems biology models and advance the development of optimized biomass feedstocks for renewable fuels and chemicals.

**Supplementary Information:**

The online version contains supplementary material available at 10.1186/s13068-023-02287-2.

## Background

The analysis of metabolites in lignocellulosic biomass is important for the production of sustainable feedstocks that will serve as a renewable source of fuels and chemicals. Metabolite content and composition in biomass are impacted by and provide insights related to genetics, carbon flux, environmental stress responses, and disease resistance. For example, *Populus* species, a promising woody feedstock for the production of renewable materials whose genome has been fully sequenced [1], produce secondary metabolites in leaves that vary genetically and according to stresses and environmental responses [2–5]. A comprehensive understanding of *Populus* leaf metabolomics can provide meaningful insight regarding plant biology and physiology and be leveraged for the domestication and rational design of *Populus* feedstocks to ensure crops with highest biomass yield and optimal composition, as well as disease resistance and environmental stress tolerance.

In order to perform genome-wide association studies (GWAS), develop systems biology models and produce superior biomass feedstocks with many favorable characteristics, large sample populations of naturally varying genotypes and phenotypes must be studied. The measurement of certain phenotypes in large sample populations can be cumbersome if high-throughput methodologies are not available. High-throughput methods using unmanned aerial vehicles (UAV) have been developed for the measurement of growth traits in plants and rapid biomass characterization methods have been established for estimation of lignin and sugar content and enzymatic recalcitrance [6, 7]. Comprehensive secondary metabolite profiles of biomass samples are difficult to achieve in high-throughput pipelines due to the number of steps required to prepare, extract and analyze samples. Typically, biomass samples are homogenized, extracted with solvent, with metabolites potentially needing to then be derivatized prior to undergoing chromatography prior to detection, identification, and quantitation of individual metabolite components. While comprehensive metabolomics relies on highly resolved and unbiased methods capable of distinguishing and quantifying specific metabolites, these methods are not necessarily conducive to large sample set analysis in a reasonable timeframe, particularly if there are many metabolites present in the extracts. There have been a number of high-throughput metabolic screening methods developed for plant metabolomics, particularly involving the use of NMR [8] advanced mass spectrometry [9] and colorimetric [10] approaches to analyze extracts. Also, the rapid determination of biomass cell wall polymer content and composition has been achieved using minimal sample preparation followed by analysis using near-infrared spectroscopy or pyrolysis-Molecular Beam Mass Spectrometry [7]. However, high-throughput methods used to screen or estimate plant metabolite profiles prior to in-depth metabolite characterization, particularly with minimal sample preparation or without extraction methodology, are lacking. Rapid screening techniques for plant metabolomics could provide insight that would alleviate pressure on comprehensive pipelines by providing down-selection criteria and prioritization of samples for analysis. High-throughput metabolite screening may also provide data that could be used directly to develop plant biology systems models and advance genomics research.

Here, we report the analysis of *Populus trichocarpa* leaves using pyrolysis-Molecular Beam Mass Spectrometry (py-MBMS) as a screening technique for metabolomics analysis. Two separate sets of leaves were analyzed where one set was used to build spectroscopic strategies for metabolite predictions using GC/MS analysis of extracts and the other set was ranked according to extractable aromatic metabolite composition and validated using GC/MS analysis of extracts. PLS models were also constructed from the two sets of leaves to demonstrate the ability to use py-MBMS data to predict extractable aromatic metabolite content in extract when a priori metabolite data are available within an analysis set; as well as to show differences in metabolic profiles across different sample sets. Greater than 250 milled and lyophilized whole biomass samples can be analyzed daily by the simplified py-MBMS screening method without any additional sample preparation (in comparison to GC/MS which requires extraction of milled leaf material, dry-down and derivatization of extract followed by dilution). Additionally, Py-MBMS simultaneously provided estimations of lignin content and monolignol composition in the leaf samples.

## Results

### Metabolite profiles of *P. trichocarpa* leaves

Metabolites present in *P. trichocarpa* leaves as determined by GC/MS analysis of extract are detailed in Additional file 1: Table S1. Over 100 metabolites were identified and semi-quantified relative to an internal standard by GC/MS analysis of extract from the set collected from Clatskanie, OR and of those metabolites, 80 were classified to be “aromatic” in nature (consisting of phenolics, benzoates, salicylic acid moieties, etc.). Metabolites were also identified and semi-quantified in the second set of extracts, leaves collected from Boardman, OR, based on GC/MS analysis, of these 49 were positively identified to be aromatic.

### Py-MBMS analysis of *P. trichocarpa* leaves

Py-MBMS analysis of the set of leaves from *P. trichocarpa* genotypes grown in Clatskanie, OR was used to analyze spectral features consistent with biomass composition that would enable estimation of the relative abundance of specific metabolites and metabolite classes present in the leaves; and is also capable of estimating relative lignin content and syringyl/guaiacyl (S/G) ratio. Table 1 shows the average lignin content and S/G ratio of the leaves determined by summation of mean-normalized ion intensities of *m/z* 120, 124 (G), 137 (G), 138 (G), 150 (G), 152, 154 (S), 164 (G), 167 (S), 168 (S), 178 (G), 180, 181, 182 (S), 194 (S), 208 (S) and 210 (S), where G denotes primarily guaiacyl-derived ions, S denotes primarily syringyl-derived ions, and other ions either derive from other lignin monomers or multiple sources.

**Table 1:** Lignin content and composition of *P. trichocarpa* leaf sets as estimated by py-MBMS

*P. trichocarpa* leaf set	Lignin content (DW %)	S/G
Clatskanie (*n* = 219)	9.7 (± 0.7)	0.6 (± 0.1)
Boardman (*n* = 223)	9.4 (± 0.6)	0.8 (± 0.1)

**Fig. 1:**
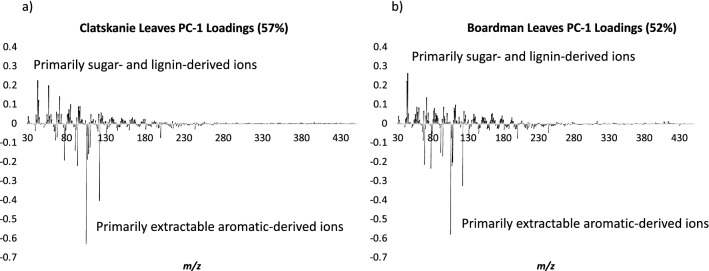
Spectral loadings (PC-1) from principal component analysis of a.) Clatskanie set and b.) Boardman set of *P. trichocarpa* leaves

The leaves in both sets exhibit significantly lower lignin content and S/G as is expected and compared to that typically seen in mature woody stem xylem tissue [11–14]. Additionally, the variation in ions and principal component analysis (PCA) show that the lignin-derived ions are not the main source of spectral (and hence compositional) variance across the sets. Principal component analysis (PCA) loadings for the first principal component plotted in spectral format to demonstrate the variation in the Clatskanie leaf set arises primarily from ions *m/z* 43, 57, 71, 85, 95, 97, which originate primarily from sugars, as well as *m/z* 77, 91, 94, 105, and 122, derived primarily from aromatics [15, 16], which were generally negatively correlated with the sugar-derived ions (Fig. 1a). Aside from these ions having generally opposing signs (orthogonal in other rotations) in PCA spectral loadings, Pearson correlation coefficients (PCC) showed negative correlations; for example for *m/z* 57 and *m/z* 77 in the Clatskanie set have PCC of − 0.64 and PCC between *m/z* 85 and *m/z* 94 in the Boardman set is -0.60.

Additionally, most of spectral variation originates from similar ions derived from aromatics, including *m/z* 68, 71, 91, 94, 105, 107, 108, and 122 (Fig. 1b) in the Boardman leaf set. However, various minor differences from a number of sources (true biomass compositional variation, instrumental drift, etc.) make it difficult to directly compare the two sets and develop PLS modeling approaches that can be used across sets without including several of the same standards, samples, etc. in each set. In order to circumvent this without requiring a priori metabolomics by GC/MS, and because these differences may not be the same for any future sets being compared, and also to simplify future analyses, the main source of spectral variation (being ions derived from aromatics) was used as a rough estimate to compare samples within the sets in the same way lignin content has been compared using this method over the last decade [7, 11, 12, 14, 17–19].

### Aromatic metabolite predictions using py-MBMS

**Fig. 2:**
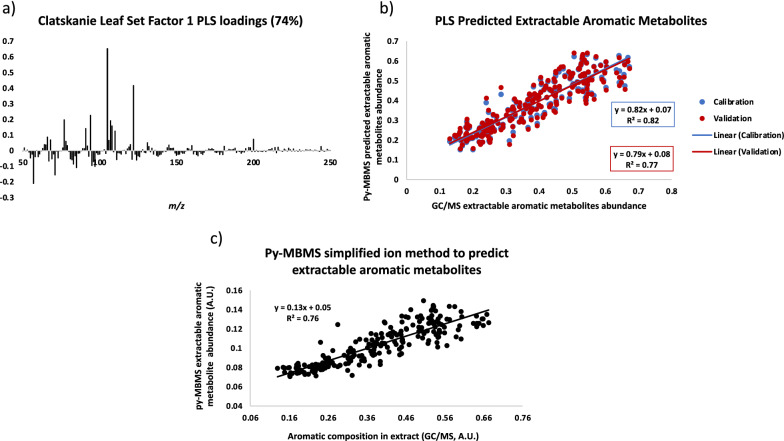
PLS model constructed from Py-MBMS spectra and GC/MS data of Clatskanie set of *P. trichocarpa* leaves. **a** Spectral loadings, **b** calibration and validation for prediction of extractable aromatic metabolites in Clatskanie set of *P. trichocarpa* leaves using PLS model, **c** Py-MBMS simplified ion method predictions of extractable aromatic metabolites relative to GC/MS analysis

**Fig. 3:**
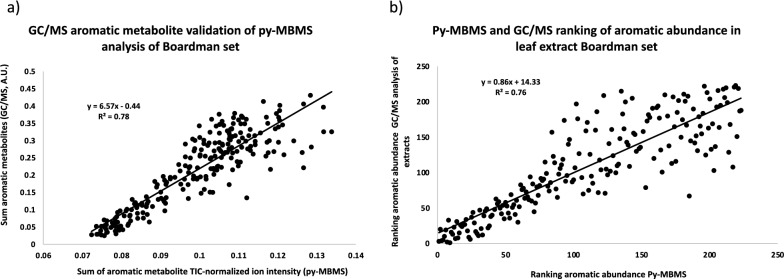
**a** Validation of Py-MBMS analysis using the simplified ion method for sum of aromatic metabolites in Boardman set of *P. trichocarpa* leaves by GC/MS. **b** Correlation of ranking of aromatic metabolite abundance in Boardman set of *P. trichocarpa* leaves by GC/MS and py-MBMS analysis

Pearson correlation coefficients were determined for select ions in the MBMS spectra, lignin content and monolignol ratios (S/G), as well as the total fractional abundance of extractable aromatics determined by GC/MS from the Clatskanie set of leaves (Table 2). The select ions in the MBMS spectra (Table 2) and the ions chosen to represent the sum of extractable aromatic metabolites were chosen based on the correlation coefficients with extractable aromatic metabolites, the principal component analyses (Fig. 1), and the loadings of the ions in the PLS models constructed using this data (Fig. 2). The PLS models used to predict the relative aromatic composition of extractable metabolites were primarily driven by the ions known to derive from aromatic species [15], primarily *m/z* 68, 71, 77, 91, 94, 105, 107, 108, and 122, which were also the main source of variation in the spectra of the set and provided a reasonable estimation and validation of using py-MBMS to predict aromatic metabolite composition in poplar leaf extracts. The sum of the aromatic metabolite ion intensities in the MBMS spectra from the Boardman set had a correlation coefficient with the sum of the extractable aromatic metabolites determined by GC/MS (sum of GC/MS peak areas normalized to internal standard from aromatic species, see Additional file 1: Table S1) of 0.87 and *R*^2^ of 0.78 (Fig. 3a). This simplified method of summing the ion intensities (*m/z*) 68, 71, 77, 91, 94, 105, 107, 108, and 122 to predict the relative abundance of aromatic metabolites in extract could theoretically be used in place of PLS modeling for ranking samples when limited a priori knowledge of metabolite information is available.

**Table 2:** Pearson correlation coefficients between select ions from MBMS spectra of *P. trichocarpa* leaves from Clatskanie, OR and select metabolites as determined by GC/MS (based on area count) as well as lignin content, S/G ratios and the summation of metabolite-derived ions from MBMS spectra and the total aromatic metabolites in extract determined by GC/MS

Trait	Lignin content	S/G	Sum metabolite ions in MBMS^a^	Aromatics by GC/MS
*m/z* 64	− 0.34	0.02	0.68	0.65
*m/z* 65	− 0.38	0.32	0.74	0.69
*m/z* 66	− 0.54	0.32	0.87	0.80
*m/z* 68	− 0.49	0.30	0.71	0.59
*m/z* 77	− 0.64	0.38	0.98	0.85
*m/z* 78	− 0.43	0.23	0.72	0.66
*m/z* 91	− 0.39	0.25	0.68	0.59
*m/z* 92	− 0.32	0.25	0.61	0.53
*m/z* 94	− 0.55	0.35	0.93	0.85
*m/z* 105	− 0.66	0.35	0.98	0.85
*m/z* 106	− 0.52	0.40	0.77	0.68
*m/z* 107	− 0.59	0.36	0.96	0.82
*m/z* 108	− 0.65	0.45	0.95	0.84
*m/z* 110	− 0.26	− 0.06	0.61	0.60
*m/z* 122	− 0.65	0.39	0.98	0.86
*m/z* 181	− 0.19	0.38	0.59	0.55
2′-O-salicyloylsalicin	− 0.36	0.26	0.52	0.63
2,6-cyclohexadiene-1,2-diol	− 0.45	0.29	0.69	0.55
6-hydroxy-2-cyclohexenone alcohol	− 0.52	0.38	0.80	0.71
α-salicyloylsalicin	− 0.55	0.40	0.87	0.88
benzoyl-gentisyl alcohol	− 0.34	0.29	0.54	0.50
benzoyl-salicyloylsalicin	− 0.44	0.33	0.62	0.72
benzyl-coumaroyl-glucoside	− 0.53	0.31	0.76	0.74
catechol	− 0.53	0.36	0.80	0.77
coumaroyl-tremuloidin	− 0.45	0.25	0.67	0.65
phenethyl-tremuloidin	− 0.45	0.23	0.65	0.63
salicortin	− 0.50	0.33	0.83	0.81
benzyl-salicylic acid-2-O-glucoside	− 0.40	0.30	0.66	0.75
salicylic acid	− 0.45	0.25	0.68	0.61
salicyloyl-coumaroyl-glucoside conjugates	− 0.58	0.37	0.85	0.86
salicyltremuloidin	− 0.45	0.30	0.68	0.83
salireposide	− 0.42	0.26	0.68	0.67
sum metabolite ions in MBMS^a^	− 0.66	0.39	1.00	0.87
aromatics by GC/MS	− 0.54	0.32	0.87	1.00
tremulacin	− 0.52	0.38	0.77	0.85
tremuloidin	− 0.40	0.24	0.56	0.55
trichocarpin	− 0.43	0.32	0.68	0.67

^a^Metabolite ion intensities summed 68, 71, 77, 91, 94, 105, 107, 108, and 122

While many of the extractable aromatic metabolites had a positive correlation with the sum of the aromatic metabolite intensities from the MBMS, they all differed in value and many of the signature ions produced from each metabolite overlap, making it difficult to differentiate, resolve and quantify the specific species present (Table 2). Therefore, this method was developed to rapidly estimate the total relative abundance of aromatic metabolites present in poplar leaf extract samples. Further deconvolution and speciation of specific metabolites will be the subject of future investigations.

The total aromatic fraction of secondary metabolites in the set from Boardman, OR, were predicted based on the simplified py-MBMS ion summation method and validated by GC/MS analysis of the extract. After GC/MS metabolic profiles were generated, PLS models of the Boardman set were also built to further validate the use of the simplified ion method. There is a correlation between the total abundance of the aromatic metabolites as determined by GC/MS and the sum of the aromatic metabolite ions determined by MBMS (Fig. 3a), with an *R*^2^ of 0.78 and Pearson correlation coefficient of 0.88 (Table 3) for the Boardman set of leaves, indicating that this simplified method is reasonably capable of predicting the relative abundance of aromatics in poplar leaf extracts across data sets.

**Table 3:** Correlations between select ions from MBMS spectra of *P. trichocarpa* leaves in the set from Boardman, OR and select metabolites as determined by GC/MS as well as lignin content, S/G ratios and the summation of metabolite-derived ions from MBMS spectra and the total aromatic metabolites determined by GC/MS

Trait	Lignin content	S/G	Sum metabolite ions in MBMS^a^	Aromatics by GC/MS
*m/z* 122	− 0.15	0.10	0.96	0.87
*m/z* 108	− 0.05	0.06	0.86	0.87
*m/z* 107	− 0.03	0.00	0.93	0.87
*m/z* 106	− 0.10	0.11	0.88	0.82
*m/z* 105	− 0.15	0.08	0.95	0.81
*m/z* 94	− 0.04	0.02	0.74	0.69
*m/z* 91	− 0.04	− 0.01	0.54	0.51
*m/z* 78	− 0.07	0.01	0.79	0.68
*m/z* 77	− 0.12	0.07	0.97	0.87
*m/z* 68	− 0.07	− 0.05	0.74	0.58
*m/z* 66	− 0.07	0.01	0.72	0.63
1,2,3-benzenetriol	− 0.11	0.06	0.43	0.50
1,2,4-benzenetriol	− 0.09	0.05	0.55	0.64
2,5-dihydroxybenzoic acid-2-O-glucoside	− 0.01	0.00	0.48	0.51
aromatics by GC/MS	− 0.13	0.09	0.88	1.00
benzoyl-salicyloylsalicin	− 0.12	0.06	0.67	0.49
catechol	− 0.03	0.02	0.52	0.62
phenol	− 0.06	− 0.01	0.64	0.68
salicin	− 0.01	0.03	0.61	0.72
salicyl alcohol	− 0.09	0.07	0.36	0.41
salicyl-coumaroyl-glucoside conjugates	0.04	− 0.11	0.70	0.62
salicylic acid	− 0.04	0.00	0.63	0.44
salicyltremuloidin	− 0.10	0.05	0.68	0.46
sum metabolite ions in MBMS^a^	− 0.13	0.05	1.00	0.88
tremulacin	− 0.12	0.03	0.63	0.47
tremuloidin	− 0.18	0.16	0.55	0.78
trichocarpin	− 0.06	− 0.01	0.49	0.50
trichocarpin conjugate	− 0.11	0.07	0.71	0.69
trichocarpinene	− 0.09	0.00	0.50	0.48

^a^Metabolite ion intensities summed 68, 71, 77, 91, 94, 105, 107, 108, and 122

Additionally, similarities in correlations between the two data sets for py-MBMS ions, metabolites and other compositional features include positive correlations between aromatic-derived ions, such as 105 and 122, with many salicylate metabolites (Table 3). The value of correlation coefficients for different traits differed to some degree between sets though, due, in part, to the differences in the actual metabolites detected and analyzed by GC/MS in the two sets. While these differences and metabolites themselves could not be speciated amongst these two sets, it was still possible to reasonably predict total extractable aromatic metabolites in the two sets using the simplified py-MBMS ion summation method as demonstrated in Fig. 3a. Additionally, the relative abundance of aromatic metabolites based on ranking GC/MS abundance reasonably correlates with the ranking predicted by py-MBMS (Fig. 3b).

**Fig. 4:**
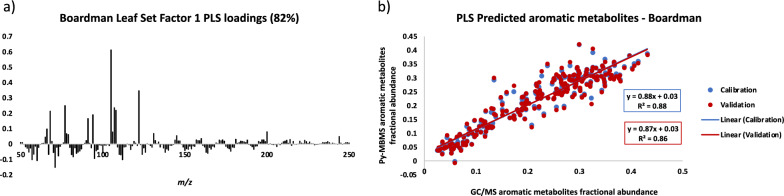
**a** Factor-1 loadings for PLS model constructed using Py-MBMS data from Boardman set of *P. trichocarpa* leaves. **b** Calibration and validation prediction of aromatic metabolites in Boardman set of *P. trichocarpa* leaves using respective PLS model

The PLS model for the set from Boardman, OR (Fig. 4) was driven by similar aromatic-derived ions as the Clatskanie set, but was different enough that the PLS models developed by one set could not be used to accurately predict aromatics in the other set. Interestingly, the PLS model for the Boardman set performed better than the model developed for the Clatskanie set. These results further validate that PLS models would be similar, but not directly translatable between different data sets and hence the need for the simplified ion intensity summation approach for screening purposes.

## Discussion

The variation in sugars vs aromatics in poplar leaves is different, but parallel to that seen in woody tissue where sugar and lignin (also aromatic, primarily phenolic) abundances are negatively correlated and the main drivers of compositional variation [11, 12]. The leaf aromatics can be distinguished from lignin-derived phenolics because higher molecular weight, known lignin-derived ions, such as *m/z* 124, 137, 154, 167, 180, 210 [7] are otherwise not strongly correlated and/or are negatively correlated with the aromatic ions derived from leaves, and therefore, *m/z* 68, 71, 77, 91, 94, 105, 107, 108, and 122 were considered as primarily originating from secondary aromatic metabolites. The use of *m/z* 68, 71, 77, 91, 94, 105, 107, 108, and 122 to estimate the relative abundance or ranking of aromatic components in samples across two different sets of almost 500 poplar leaf samples demonstrates a simple and robust method to screen samples for rapid secondary metabolite insight and for prioritization of samples for in-depth metabolomics pipelines .

## Conclusions

We have reported a rapid technique that can be used to analyze and screen biomass tissue for the relative abundance of total aromatic metabolites. Error associated with this method could result from the relative abundance of different aromatic metabolites, and further studies and computational advances could potentially enable deconvolution of spectral features to provide resolved abundances of specific metabolites. If a priori metabolic information is available for standards or a range of samples within given data sets, PLS models specific to that data set could also be used to predict relative aromatic metabolite abundances. These methods could be used to screen large populations of biomass for aromatic metabolites to inform GWAS analyses, quantitative trait loci mapping, and development of metabolomic and systems biology models. The metabolite information obtained may also be used to inform sustainability metrics and gene x environment interactions in biomass studies. Rapid metabolomic analyses would ultimately enable the rational design of sustainable biomass used for biorefinery and bioenergy applications.

## Methods

### Collection and Preparation of Leaf Samples

Leaves from 851 *P. trichocarpa* genotypes established in a common garden at Clatskanie, OR in 2009 [20, 21] were sampled over a 3-day period in July 2012, as described elsewhere [22], from which 219 samples were selected for inclusion in the present study and were selected as they varied in total and specific aromatic metabolite concentrations based on GC/MS analysis of foliar extracts. Additionally, 60 *P. trichocarpa* genotypes growing in a drought stress trial at Boardman, OR were sampled on July 11 and 12, 2018, including 2 replicate trees per genotype in a well-irrigated plot (44.7 cm in each of the 2017 and 2018 growing seasons) and 2 replicate trees growing in a drought stress plot receiving 60% (26.8 cm) the irrigation level of the well-irrigated plot. Trees were established in the spring of 2016, with the irrigation manipulation applied during the second (2017) and third (2018) growing seasons. In summary, the Boardman set consisted of 223 *P. trichocarpa* similar leaf samples collected from trees growing under different irrigation conditions at Boardman, OR in the summer of 2018 in their third year. Similar to the leaves sampled at Clatskanie, OR, a fully expanded leaf of leaf plastochron index 9 ± 1 was rapidly collected, placed on dry ice, shipped back to Oak Ridge National Laboratory, stored at -80ºC until being processed for metabolite analyses.

With Clatskanie, OR, being a mesic site in the Columbia River Delta with a high water table, trees were grown without supplemental irrigation. With Boardman, OR, typically only receiving approx. 18 cm of rainfall annually, trees were supplemented with irrigation water. Surprisingly, there were no significant differences in the total metabolite concentrations between drought-stressed and well-watered trees within a given genotype (data not shown).

### Extraction and analysis of leaf metabolites by GC/MS

For leaf samples collected at Clatskanie, OR, metabolite extraction and analysis were prepared as described previously [2, 23]. For leaf samples collected from genotypes at Boardman, OR, metabolite extraction and analysis were performed based on protocols also described previously [24]. Briefly, lyophilized samples were ground and extracted twice overnight with 2.5 mL portions of 80% ethanol. Sorbitol (75 µL; 1 mg mL^−1^) was added before extraction as an internal standard. Following centrifugation, a 500 µL aliquot of the combined extract was dried in a stream of nitrogen and silylated by addition of 500 µL of acetonitrile (TS-20062; ThermoFisher) and 500 µL of *N*-methyl-*N*-trimethylsilyltrifluoroacetamide (MSTFA) with 1% trimethylchlorosilane (TMCS) (TS-48915; ThermoFisher) and heating for 1 h at 70 °C to generate trimethylsilyl (TMS) derivatives. After 2 days, a 0.1 or 1 µL aliquot was injected into an Agilent Technologies Inc. (Santa Clara, CA, USA) 7890A gas chromatograph coupled to a 5975C inert XL mass spectrometer configured [25], and aromatic metabolites were identified, quantified, and normalized as previously described [2, 25].

The fraction of the aromatic metabolites in the dry weight (DW) extract of leaves was obtained by summing the abundance of each aromatic metabolite (μg or ng/g DW internal standard equivalent) and dividing by the total sum of the metabolites accounted for (DW internal standard equivalent) in each sample.

### Pyrolysis-MBMS

A Frontier PY2020 unit pyrolyzed 4 mg of cryo-milled biomass at 500 °C for 30 s in 80 µL deactivated stainless steel cups. In comparison to the GC/MS method, the only preparation required for py-MBMS analysis is the cryo-milling of the samples and adding the leaf samples to the MBMS cups. Each biomass sample was analyzed in duplicate. An Extrel Super-Sonic MBMS Model Max 1000 was used to collect mass spectra (*m/z* 30–450 at 17 eV) which was processed using Merlin Automation software (V3). Spectral ion intensities were normalized based on summation of total ion intensities (TIC) and spectra were subsequently analyzed using The Unscrambler X v10 (Camo, Trondheim, Norway). Lignin content was estimated by using a single point response factor relative to a representative NIST standard of known Klason lignin content using the same ions. Syringyl-to-guaiacyl (S/G) ratios were estimated by dividing the sum of S-based ions by the sum of G-based ions using mean-normalized ion intensities.

Based on the analysis of the Clatskanie set, ions (*m/z*) 68, 71, 77, 91, 94, 105, 107, and 122 were highly correlated to the abundance of aromatic metabolites and were used to predict the relative abundance of total aromatic metabolites that would have been present in the extracts of the Boardman set. Principal Component Analysis (PCA, NIPALS algorithm) was performed on mean-centered, TIC-normalized MBMS spectra resulting in 6 principal components for the Clatskanie samples explaining 57% variance and 7 principal components for the Boardman samples explaining 52% variance. Partial Least Squares (PLS) regression models constructed from the GC/MS and py-MBMS data (restricted to *m/z* 50–250, mean centered) of the two different sets were generated using the Unscrambler X V.10.5 (Camo Software) NIPALS algorithm. PLS cross validation was performed using 20 random samples, all variables were weighted equally. 3-Factor models were used for the prediction of the soluble, extractable aromatics in the Clatskanie samples and 4-Factor models were used for the prediction of the soluble, extractable aromatics in the Boardman poplar leaf samples.

## Supplementary Information

**Additional file 1.** GC-MS data from analysis of extracts from Boardman and Clatskanie poplar leaves.

## Data Availability

The Department of Energy will provide public access to these results of federally sponsored research in accordance with the DOE Public Access Plan (http://energy.gov/downloads/doe-public-access-plan). Data not presented in the manuscript is available upon request from the coauthors.
